# Understanding reasons for initiation and experience with tirzepatide among individuals with obesity or overweight: Results from the PERCEPTIONS survey

**DOI:** 10.1016/j.obpill.2026.100288

**Published:** 2026-06-17

**Authors:** Theresa Hunter Gibble, Harriet Makin, Claire Gerber, Catherine Bottomley, Xuanyao He, Sara Pisani, Michael Shepherd, Elizabeth Collins, Angela Golden, Harold Bays

**Affiliations:** a Eli Lilly and Company, Indianapolis, IN, USA; b Clarivate, London, UK; c The Obesity Society, Rockville, MD, USA; d Monroe Biomedical Research; Louisville Metabolic and Atherosclerosis Research Center, Louisville, KY, USA

**Keywords:** Real-world survey, Tirzepatide, Patient perspectives, Quality of life

## Abstract

**Background:**

Obesity contributes to multiple complications, increased mortality, and reduced health-related quality of life (HRQoL). Tirzepatide demonstrated sustained weight reduction and improvements in HRQoL in the SURMOUNT studies. Limited data are available regarding real-world experiences of participants initiating tirzepatide.

**Methods:**

This manuscript reports baseline data from the PERCEPTIONS survey, a real-world, observational study conducted in the US (June–November 2025). The study enrolled obesity medication (OM)-eligible adults with obesity (BMI ≥30 kg/m^2^) or overweight (BMI ≥27 kg/m^2^ with ≥1 obesity-related complication) and without type 2 diabetes mellitus. The survey was completed within 4–8 weeks of first tirzepatide dose. Questions included demographic and clinical characteristics, previous treatments, patient-reported outcomes, motivators/barriers to treatment initiation, and ease of tirzepatide use.

**Results:**

The descriptive study included 518 participants (mean [SD] age: 46.0 [11.58] years, 78.2% female, 69.5% White, mean [SD] BMI: 38.4 [8.38] kg/m^2^). The mean [SD] duration between the first tirzepatide dose and baseline survey completion was ∼26 (16) days; 89.2% initiated tirzepatide at 2.5 mg dose and 12.7% had previously received semaglutide. Nearly half had previously been unable to reduce weight/maintain weight reduction using exercise and dietary interventions. Participants reported substantial impairments in physical functioning, psychosocial well-being, and high internalized weight bias at tirzepatide initiation. Primary motivators for initiating tirzepatide included improving overall health and well-being (83.8%), physical functioning (58.3%), and self-confidence and/or appearance outside of work (51.4%). Common barriers to tirzepatide initiation included concerns about treatment cost (57.7%), and health insurance coverage (90%). Majority of auto-injector (98.0%) and vial users (95.8%) reported that tirzepatide was easy to use.

**Conclusions:**

This real-world survey emphasized the multifaceted disease burden experienced by participants with obesity or overweight. Participants initiating tirzepatide were highly motivated to lose weight, improve their well-being, and self-confidence. Identifying common barriers to OM access will help optimize health strategies for obesity management.

## Highlights

• Participants reported high rates of internalized weight bias and low physical functioning and psychosocial well-being scores at the time of tirzepatide initiation.
• The primary motivators for initiating tirzepatide were to improve overall health and well-being, physical functioning, and self-confidence.
• Barriers such as out-of-pocket cost and uncertainties related to insurance coverage highlight the need to address additional systemic barriers that limit equitable access to effective treatment options.

## Graphical abstract

**Figure:**
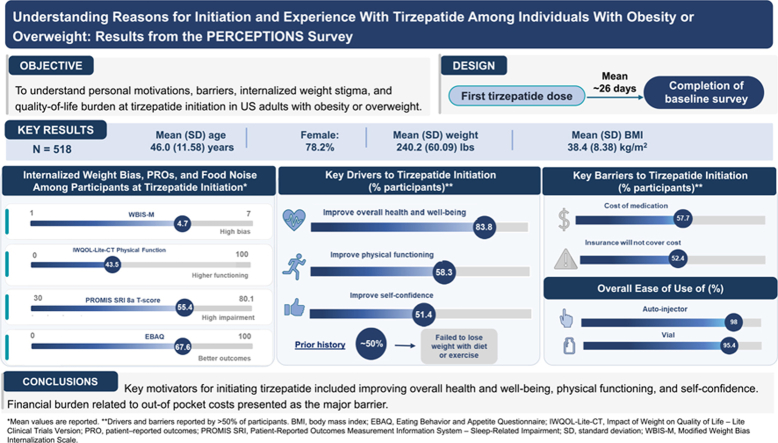

## 1 Introduction

Obesity is a chronic, relapsing disease with a global prevalence estimated at 890 million adults [1]. By the year 2030, it is predicted that obesity and overweight will affect approximately 3 billion adults – about half of the global adult population [[1], [2], [3]]. In the United States, obesity prevalence reached 40.3% between 2021 and 2023, reflecting a significant public health concern [2]. Further, obesity is associated with several chronic diseases, such as cardiovascular diseases, type 2 diabetes (T2D), and cancers, significantly increasing the risk of morbidity, mortality, and reduced life expectancy [1,[4], [5], [6]].

Although lifestyle modifications have long been considered the backbone of obesity treatment, achieving sustained weight reduction and long-term adherence are often challenging [5]. Clinical guidelines recommend the use of pharmacotherapy in conjunction with lifestyle interventions for sustained weight reduction and improving health-related quality of life (HRQoL) [7,8]. Tirzepatide is a once-weekly glucose-dependent insulinotropic polypeptide (GIP) and glucagon like peptide-1 (GLP-1) receptor agonist approved for weight management [9]. The Phase 3 SURMOUNT 1–4 trials demonstrated that tirzepatide treatment resulted in substantial and clinically meaningful weight reductions versus placebo in participants with obesity or overweight [[10], [11], [12], [13]]. Tirzepatide treatment was further associated with improved self-reported HRQoL outcomes, including physical and psychosocial functioning, across the SURMOUNT trials [[14], [15], [16], [17]].

Despite robust clinical evidence supporting the positive impact of tirzepatide on patient lives [18], comparatively less is known about the real-world experiences of participants initiating this therapy. Understanding motivations of participants for initiating tirzepatide as well as perceived barriers is critical for obesity management as these factors can significantly influence individual behaviours, treatment adherence, and overall health outcomes.

This non-interventional, real-world survey assessed the motivations and barriers for tirzepatide initiation among participants in the US with obesity or overweight and evaluated internalized weight bias and quality of life among participants at the time of tirzepatide initiation.

## 2 Methods

### 2.1 Study design

This observational, real-world study utilized baseline data from the PERCEPTIONS survey conducted from June to November 2025 in the US. The PERCEPTIONS survey was developed and conducted by Clarivate, in collaboration with Eli Lilly and Company, and was co-designed with input from both patients with obesity or overweight and healthcare providers (HCPs). The PERCEPTIONS survey included a combination of bespoke questions on demographics and clinical characteristics, prior treatment experience, motivations for initiating tirzepatide, treatment goals, as well as validated patient-reported outcome (PRO) questionnaires. The content and language of the survey was reviewed and piloted by participants with obesity or overweight before survey launch to assess the interpretation of survey instructions, questions, and response options. Specific recall period was assigned to study measures, as outlined below.

Participants were recruited through clinical sites in the US and specialist medical recruitment agencies. Institutional Review Board-approved recruitment text and screener forms were used for recruitment activities.

The study included obesity medication (OM)-eligible adults (≥18 years) with obesity (BMI ≥30 kg/m^2^) or overweight (BMI ≥27–29.9 kg/m^2^ with ≥1 obesity-related complication) who were initiating tirzepatide treatment. Eligible participants were employed full-time in the US at the time of tirzepatide initiation for the treatment of obesity or overweight. Participants with a diagnosis of type 2 diabetes mellitus, previous bariatric surgery, prior use of tirzepatide, or participation in clinical trials involving tirzepatide or other obesity-related molecules were excluded.

### 2.2 Study measures

**Fig. 1:**
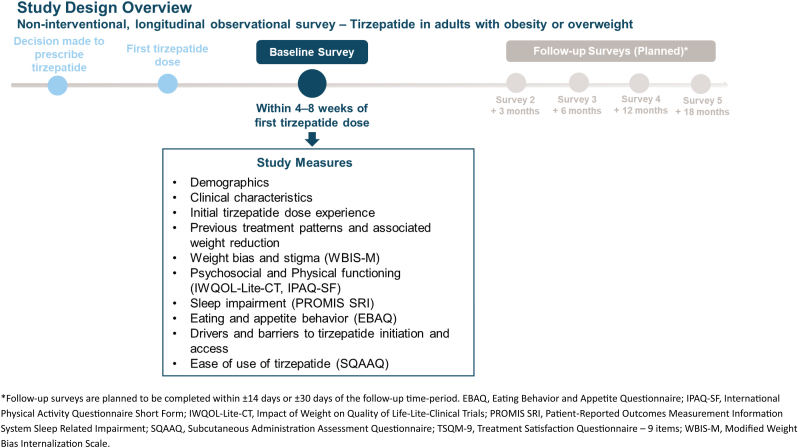
PERCEPTIONS survey design.

Each participant completed their baseline survey within 4–8 weeks of receiving the first tirzepatide dose (Fig. 1). Baseline demographic and clinical characteristics including age, sex, race, ethnicity, employment duration, highest level of education, weight, BMI, and obesity-related comorbidities (ORCs) were collected. Initial tirzepatide dose experience data was collected, including assessment of dosage, and device used for administration of first tirzepatide dose, as well as use of telehealth services, and prescribing HCP. Previous treatment patterns and associated weight reduction were evaluated based on prior use of OMs and experience with behavioral and lifestyle modification strategies. Weight bias and stigma were assessed using the 10-item Modified Weight Bias Internalization Scale (WBIS-M). Responses were scored on a 7-point Likert scale (“Strongly Disagree” to “Strongly Agree”) with higher scores indicating greater internalized weight bias [19,20]. The Impact of Weight on Quality of Life (IWQOL-Lite-CT), a 20-item PRO instrument was also included to evaluate the impact of body weight on quality of life in adults with obesity. It comprises domains of Physical, Physical Function, Psychosocial Self-esteem, and a Total score. Items are rated on a 5-point Likert scale (“never” to “always”) or 5-point truth scale (“not at all true” to “completely true”), with higher scores indicating a higher level of functioning [21,22]. The International Physical Activity Questionnaire (IPAQ-SF) assesses physical activity levels focusing on frequency and duration of walking, and moderate- and vigorous-intensity activities over the past seven days [23]. Activity data were measured in minutes, converted to metabolic equivalent of task (MET) minutes, and participants were categorized as low, moderate, or highly active. Sleep-related impairment was assessed using the Patient-Reported Outcomes Measurement Information System (PROMIS) Sleep-Related Impairment (SRI) Short Form 8a. This measure assesses alertness, sleepiness, and tiredness during usual waking hours on a 5-point Likert scale (“Not at all” to “Very much”) in the past 7 days. Higher scores indicate greater sleep-related impairment [24,25]. Food noise was evaluated using the 23-item version of the Eating Behavior and Appetite Questionnaire (EBAQ), which assesses appetite, eating control, and triggers on a 5-point Likert scale (“never” to “always”) in the past 7 days. Multiple items were grouped into distinct scoring domains (appetite and eating behavior), and total score, with higher scores indicative of a well-controlled appetite and better eating habits [26,27]. Participants were also asked to select from a list of reasons to assess drivers and barriers to tirzepatide initiation, for example, “I wanted to improve my overall health and well-being”. Ease of tirzepatide use was measured by the 12-item Subcutaneous Administration Assessment Questionnaire (SQAAQ) [28]. Items were scored on a 7-point Likert scale (“strongly disagree” to “strongly agree”), with higher scores indicating greater ease of use. *Agree*, *slightly agree*, or *strongly agree* were combined to calculate ease of use. No recall period was provided.

### 2.3 Statistical analyses

The analysis population comprised all participants who completed the survey and met eligibility criteria. Data were summarized using descriptive statistics: categorical variables were presented as frequencies and percentages; continuous variables were presented as mean (standard deviation [SD]), median (interquartile ranges [IQR]), or median (minimum–maximum [Min–Max]). Data from validated PRO measures were scored in line with their published scoring manuals. Analyses used complete-case data per variable, with Ns reported in tables/figures. Missing data were presented for each question; no adjustments were made to account for missing data beyond following the scoring algorithms.

## 3 Results

### 3.1 Baseline demographics and clinical characteristics

The study included 518 participants who were employed full-time at the time of tirzepatide initiation; 368 participants were recruited through specialist recruitment agencies, and 150 were recruited through clinical sites. The mean (SD) duration between the first tirzepatide dose and survey completion was ∼26 [16] days (Table 1). The mean (SD) age of participants was 46.0 (11.58) years. The majority of participants were female (78.2%) and White (69.5%). The mean (SD) weight was 240.2 (60.09) lbs, and the mean (SD) BMI was 38.4 (8.38) kg/m^2^ (Table 1). Participants were employed for a mean duration of 89.8 months (∼7.5 years). The highest educational qualifications were bachelor's degree (29.3%) and graduate or postgraduate degree (29.2%). Common ORCs were anxiety (33.8%), obstructive sleep apnea (32.4%), and back pain (30.1%) (Table 1).

**Table 1:** Baseline demographics and clinical characteristics.

Characteristics	N = 518
**Age (years)**	
Mean (SD)	46.0 (11.58)
**Sex, n (%)**	
Female	405 (78.20)
Male	112 (21.60)
Intersex	1 (0.20)
**Race, n (%)**	
White	360 (69.50)
Black or African American	128 (24.70)
Asian	16 (3.10)
American Indian or Alaskan Native	8 (1.50)
Native Hawaiian or Other Pacific Islander	2 (0.40)
Other	23 (4.40)
**Ethnicity, n (%)**	
Hispanic or Latino	50 (9.70)
Not Hispanic or Latino	468 (90.30)
**Employment duration at current primary full-time workplace/company, months**	
Mean	89.8
Median	51.0
IQR	26.0–121.0
**Highest level of education, n (%)**	
Bachelor's degree	152 (29.30)
Graduate/Post-graduate degree	151 (29.20)
High school diploma or equivalent	117 (22.60)
Associate's degree	87 (16.80)
Some high school, no diploma	1 (0.20)
Other	10 (1.90)
**Weight (lbs)**	
Mean (SD)	240.2 (60.09)
**BMI (kg/m^2^)**	
Mean (SD)	38.4 (8.38)
**Obesity-related comorbidities, n (%)**^a^	
Anxiety	175 (33.8)
Obstructive sleep apnea	170 (32.4)
Back pain	156 (30.1)
Depression	122 (23.6)
Prediabetes	111 (21.4)
Essential primary hypertension	73 (14.1)
Asthma	72 (13.9)
GERD	65 (12.5)
Osteoarthritis (hip and/or knee)	49 (9.5)
PCOS	41 (7.9)
Insulin resistance	37 (7.1)
Dyslipidemia	35 (6.8)
Urinary incontinence	23 (4.4)
**Days between first tirzepatide dose and survey**	
N	518
Mean (SD)	25.6 (16.27)
Median	23.00
Min–Max	0.0–63.0
IQR	12.0–38.0
**Distribution of first tirzepatide doses, n (%)**	
2.5 mg	462 (89.2)
5 mg	41 (7.9)
7.5 mg	8 (1.5)
10 mg	4 (0.8)
15 mg	1 (0.2)
I don't know/I can't remember	2 (0.4)
**Device of first tirzepatide dose administration, n (%)**	
Auto-injector/single dose self-injector pen	351 (67.8)
Single dose injected from a vial/bottle	167 (32.2)
**Use of telehealth services (virtual or app-based consultation with a healthcare professional) to obtain first tirzepatide prescription, n (%)**	
No	377 (72.8)
Yes	141 (27.2)
**Prescription for first dose of tirzepatide, n (%)**	
Primary Care Physician (e.g., family doctor or internal medicine doctor)	327 (63.1)
Obesity/overweight specialist	58 (11.2)
Nurse Practitioner	49 (9.5)
Endocrinologist	20 (4.0)
Physician Associate	15 (2.9)
Other	13 (2.5)
Bariatric surgeon	10 (1.9)
Cardiologist	8 (1.5)
I don't know	8 (1.5)
Pulmonologist	4 (0.8)
Gynecologist	3 (0.6)
Psychiatrist	2 (0.4)
Gastroenterologist	1 (0.2)

a Other obesity-related comorbidities with an incidence of ≤2.3% included cancer, cardiovascular disease, female infertility, and metabolic syndrome to name a few. BMI, body mass index; GERD, gastrointestinal reflux disease; IQR, interquartile range; PCOS, polycystic ovary syndrome; SD, standard deviation.

### 3.2 Initial tirzepatide dose experience

Tirzepatide initiation most commonly occurred at 2.5 mg dose (89.2%); first dose administration was predominantly via auto-injector (67.8%), although nearly one-third of participants (32.2%) used vials. Furthermore, 27.2% participants obtained their first tirzepatide prescription through a telehealth service, such as virtual or app-based consultation with a healthcare professional. The majority (63.1%) were prescribed their first dose of tirzepatide by a primary care physician, followed by obesity/overweight specialists (11.2%) (Table 1).

### 3.3 Previous treatment patterns and weight reduction journey

Before initiating tirzepatide, 12.7% (n = 66) had previously taken semaglutide and 4.6% (n = 24) had previously taken liraglutide (Table S1). A considerable proportion of participants reported that they were previously unable to lose weight or maintain weight reduction with an exercise routine (49.6%), tracking physical activity or exercise (43.1%), consuming healthy foods and drinks (45.2%), and eating diet products (48.6%). Less than half of the participants reported participating in workplace or non-workplace wellness programs (30.3% and 27.8%, respectively) for weight reduction; approximately one-third of these reported experiencing weight reduction benefits (Table S1).

### 3.4 Weight bias and stigma

Overall, the mean (SD) WBIS-M total score was 4.7 (1.30), indicating substantial internalized weight bias (Table 2). The total scores ranged from 1.2 to 7.0, indicating moderate variability in weight bias internalization. Item-level analyses indicated the highest mean scores for wishing to drastically change one's weight (Q3; 5.9 [1.36]) and being comfortable with current weight (Q8; 5.9 [1.31]). In contrast, lower mean scores were observed for feeling undeserving of a fulfilling social life due to weight (Q7; 3.2 [1.95]) and for weight-related self-hatred (Q5; 3.7 [2.01]) (Table 2).

**Table 2:** Weight bias and stigma at baseline as assessed by WBIS-M.

	Q1. I am less attractive than most other people because of my weight.	Q2. I feel anxious about my weight because of what people might think of me.	Q3. I wish I could drastically change my weight.	Q4. Whenever I think a lot about my weight, I feel depressed.	Q5. I hate myself for my weight.	Q6. My weight is a major way that I judge my value as a person.	Q7. I don't feel that I deserve to have a really fulfilling social life, because of my weight.	Q8. I am OK being the weight that I am.	Q9. Because of my weight, I don't feel like my true self.	Q10. Because of my weight, I don't understand how anyone attractive would want to date me.	Total
Total	518	518	518	518	518	518	518	518	518	518	518
Mean (SD)	5.1 (1.67)	5.1 (1.70)	5.9 (1.36)	5.0 (1.67)	3.7 (2.01)	4.2 (1.94)	3.2 (1.95)	5.9 (1.31)	5.1 (1.69)	4.2 (2.05)	4.7 (1.3)
Median	5.00	5.00	6.00	5.00	4.00	5.00	3.00	6.00	5.00	4.00	4.80
Min–Max	1–7	1–7	1–7	1–7	1–7	1–7	1–7	1–7	1–7	1–7	1.20–7.0
IQR	4.75–6.00	5.00–6.00	5.00–7.00	4.00–6.00	2.00–5.00	2.00–6.00	1.00–5.00	5.00–7.00	4.00–6.00	2.00–6.00	3.80–5.70

Each item in WBIS-M has a range of scores from 1 to 7. Higher scores indicate worse weight bias internalization. The WBIS-M (10-item) Total Score is calculated as an average score per participant. IQR. Interquartile range; Max, maximum; Min, minimum; SD, standard deviation; WBIS-M, Modified Weight Bias Internalization Scale.

### 3.5 Psychosocial and physical functioning

Participants reported generally low IWQOL-Lite-CT scores, with a mean (SD) total score of 38.2 (20.46) and similarly low composite scores across domains (Physical Function: 43.5 [23.90], Physical: 42.3 [22.89], and Psychosocial: 36.0 [22.11]) (Table 3). Based on IPAQ classification criteria, activity levels were distributed across categories, with 31.7% of participants were classified as highly active, 32.4% as moderately active, and 21.4% as having low activity levels (Table 3). Participants reported a median of 1470 MET minutes per week (IQR: 693–3144). The mean (SD) daily sitting time was 418 (209.09) minutes (∼7 h), indicating major sedentary time (Table 3).

**Table 3:** Patient reported outcomes at baseline in individuals who initiated tirzepatide.

***IWQOL-Lite-CT scores at baseline***^a^				
	**N**	**Mean (SD)**	**Median**	**Min–Max**

**Total**	518	38.2 (20.46)	37.5	0–100
**Physical Composite**	518	42.3 (22.89)	39.3	0–100
**Physical Function Composite**	518	43.5 (23.90)	40	0–100
**Psychosocial Composite**	518	36.0 (22.11)	34.6	0–100

***Physical functioning as assessed by IPAQ-SF***^b^				
	**N**	**Mean (SD)**	**Median**	**Min–Max**

**Total Time Spent Sitting, Minutes**	444	418.0 (209.09)	420	15–960
**MET Minutes per Week**	443		1470	0–18090
**Activity, n (%)**				
**High**	164 (31.7)			
**Moderate**	168 (32.4)			
**Low**	111 (21.4)			
**Excluded**	4 (0.8)			
**Missing**	71 (13.7)			
**Total**	518 (100.0)			

***Sleep-related impairment (SRI)***^c^				
	**N**	**Mean (SD)**	**Median**	**Min–Max**

**PROMIS SRI 8a T-Scores**	518	55.4 (9.80)	56.1	30.0–80.1

a Scores are transformed to a scale of 0–100, with higher scores reflecting better levels of functioning.

b IPAQ-SF measured physical activity as time spent doing vigorous and moderate activity, walking, and sitting, and captures the time spent on each activity over the past 7 days. Activity data was analyzed in minutes and converted into METs, and participants were categorized as low, moderate, or highly active as per IPAQ scoring manual criteria. High activity: vigorous intensity activity on at least 3 days achieving a total of at least 1500 MET mins/week, OR 7 or more days of any combination of walking, moderate intensity, or vigorous intensity activities achieving at least 3000 MET mins/week. Moderate activity: 3 or more days of vigorous intensity activity of at least 20 min per day, OR 5 or more days of moderate intensity activity and/or walking of at least 30 min/day, OR 5 or more days of any combination of activities achieving at least 600 MET mins/week. Low activity: Individuals not meeting criteria for moderate or high activity.

c PROMIS-SRI assesses alertness, sleepiness, and tiredness during usual waking hours on a 5-point Likert scale in the past 7 days. The PROMIS SRI raw scores, ranging from 8 to 40, are converted to T-scores, ranging from 30.0 to 80.1 for the 8a Short Form. A T-score of 50 is the average for the United States general population with a standard deviation of 10. A higher score represents more of the concept being measured (e.g., higher score = worse sleep impairment). Scores are reported as T-scores. IPAQ-SF, International Physical Activity Questionnaire – Short Form; MET, metabolic equivalent of task; IQR, interquartile range; IWQOL-Lite-CT, Impact of Weight on Quality of Life – Lite Clinical Trials Version; PROMIS SRI, Patient-Reported Outcomes Measurement Information System – Sleep-Related Impairment; SD, standard deviation.

### 3.6 Sleep impairment

The mean (SD) PROMIS SRI T-score was 55.4 (9.80), which is marginally higher than the average score for the US general population [29], indicating mild sleep-related impairment at baseline (Table 3).

### 3.7 Eating and appetite behavior

**Fig. 2:**
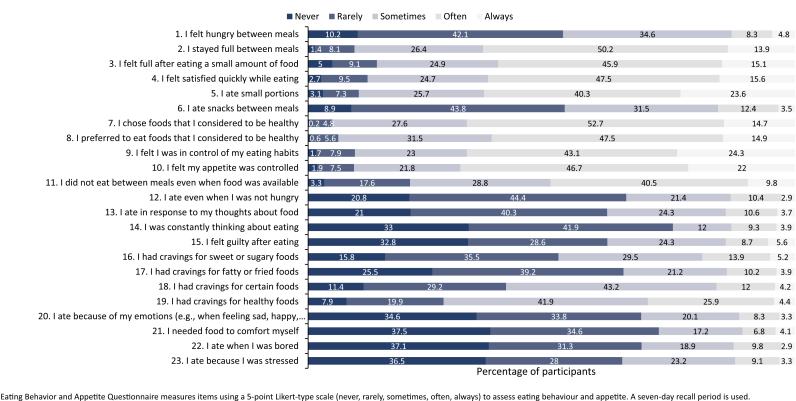
Eating Behavior and Appetite Questionnaire.

The mean (SD) EBAQ total score was 67.6 (18.64). The mean (SD) appetite domain score was 66.3 (18.84) and the mean eating behaviour domain score was 68.5 (20.72) (Table 4). The majority of participants (64.1%) felt full between meals, and 15.8% participants ate snacks between meals; 13.3% participants ate even when they were not hungry. (Fig. 2). Further, participants reported always or often craving sweet or sugary foods (19.1%) and fatty or fried foods (14.1%) Participants also reported always or often eating because they were bored (12.7%) or stressed (12.4%) (Fig. 2).

**Table 4:** Food noise as assessed by EBAQ scores.

	Appetite Domain Score (7 items)∗	Eating Behavior Domain Score (10 items)∗∗	Total Score (17 items)∗∗∗
Total	518	518	518
Mean (SD)	66.3 (18.84)	68.5 (20.72)	67.6 (18.64)
Median	67.9	72.5	72.1
Min–Max	0–100	0–100	0–100
IQR	53.57–78.57	57.50–85.00	58.82–79.41

Domain EBAQ scores range from 0 to 100, with higher scores reflecting better outcomes (reduced appetite, satisfaction with small portions, more eating control, less frequent cravings, and less emotional eating). ∗EBAQ-23 items 2, 3, 4, 5, 9, 10, and 11 loaded to the appetite domain. ∗∗EBAQ-23 items 6, 12, 13, 14, 16, 17, 20, 21, 22, and 23 loaded to eating behavior domain. ∗∗∗EBAQ-23 items 2, 3, 4, 5, 9, 10, 11, 6, 12, 13, 14, 16, 17, 20, 21, 22, and 23 were loaded to the total score domain. The recall period was 7 days. EBAQ, Eating Behavior and Appetite Questionnaire; IQR, interquartile range; Max, maximum; Min, minimum; SD, standard deviation.

### 3.8 Drivers and barriers to tirzepatide initiation and access

The most common reasons participants initiated tirzepatide were to improve their overall health and well-being (83.8%), followed by improvement in physical functioning (58.3%) and self-confidence and/or appearance outside of work (51.4%) (Fig. 3a). Nearly half of the participants (47.1%) reported that their healthcare clinician recommended that they start a medication to treat obesity/overweight. (Fig. 3a). A substantial proportion of participants delayed initiating tirzepatide treatment for obesity/overweight due to financial concerns and insurance coverage (Fig. 3b). Specifically, 57.7% were worried about the cost of medication, 52.4% were worried whether their personal health insurance would cover the treatment, and 47.3% expressed concern that their employer-provided health insurance might not include coverage for tirzepatide (Fig. 3b).

**Fig. 3a:**
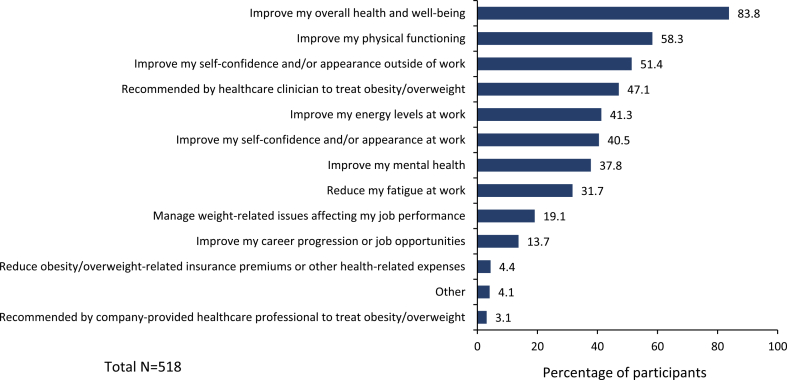
Drivers to tirzepatide initiation and access.

**Fig. 3b:**
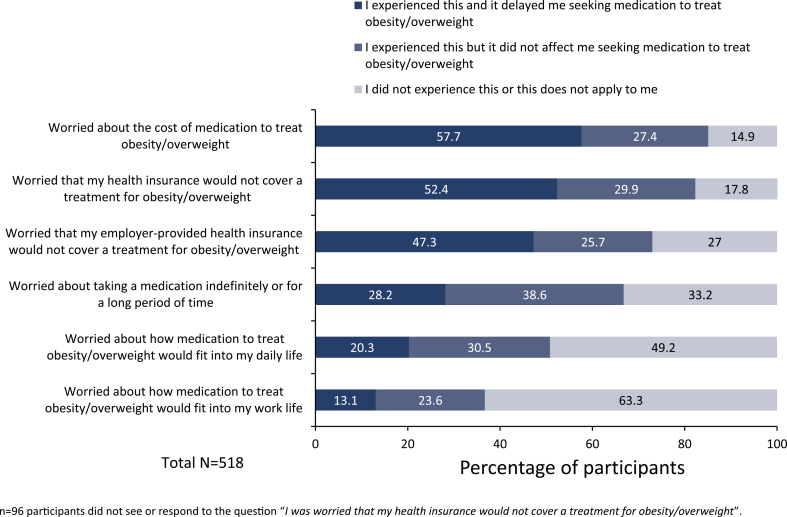
Barriers to tirzepatide initiation and access.

### 3.9 Ease of use of tirzepatide

The majority of participants reported that tirzepatide was easy to use across all SQAAQ questions (Table S2). Overall ease of use was reported by 98.0% of auto-injector users and 95.8% of vial users. Among auto-injector users, 96.3% said unlocking the pen was simple, 97.4% found injecting easy, and 94.3% could easily confirm dose completion. For vial users, 89.2% reported ease of injection and 91.6% easily confirmed dose completion. Both groups reported similar ease of storage in refrigerator (auto-injector: 97.7%, vial: 97.0%). Confidence in ability to use dose was reported by 97.4% of auto-injector users and 89.2% of vial users; 95.2% of auto-injector users and 93.4% of vial users indicated they were “confident that their dose is complete” (Table S2).

## 4 Discussion

This US real-world study provides evidence of the disease burden, motivations, and barriers associated with initiating tirzepatide treatment among participants with obesity or overweight, offering insights for effective obesity management, and improving overall health outcomes.

At baseline, participants in our study reported high rates of internalized weight bias, which has been independently associated with poorer sleep quality, reduced HRQoL, and diminished self-confidence [30,31]. They also experienced reduced physical functioning and psychosocial well-being as well as mild sleep impairment, indicating limitations across various aspects of daily living and heterogeneous baseline disease burden. These findings concur with the primary motivations reported by participants for initiating tirzepatide treatment, namely improving overall health and well-being, physical functioning, self-confidence and/or appearance outside of work. In the SURMOUNT 1–4 trials, treatment with tirzepatide versus placebo resulted in significantly greater improvements in HRQoL, particularly among participants who had limitations in physical function at baseline [[14], [15], [16], [17]]. Ongoing longitudinal survey data from this cohort will provide real-world evidence characterizing HRQoL outcomes among participants receiving tirzepatide.

Most participants in our study had not received any obesity pharmacotherapy prior to initiating tirzepatide treatment, while a considerable proportion were unable to lose/maintain weight through lifestyle interventions and exercise. These findings underscore the importance of combining lifestyle interventions with pharmacotherapy to achieve meaningful and sustained weight reduction [32]. The baseline survey was conducted within 4–8 weeks following tirzepatide initiation. The majority of participants always or often felt satisfied quickly while eating (63.1%) and reported that their eating habits and appetite were always or often under control (22–46.7%); they rarely or never had cravings for sugary or fried foods (15.8%–39.2%); and preferred eating healthy foods (62.4%) (Fig. 2). These favourable outcomes may reflect early improvement in food noise with tirzepatide treatment and are broadly consistent with evidence from clinical trials demonstrating reductions in appetite and food-related desire in the weeks following tirzepatide initiation [[33], [34], [35]]. In a randomized Phase 1 trial, participants receiving tirzepatide had reduced intake of all three macronutrients at Week 3, approximately 72% reduction in energy intake at lunch at Week 6, and reduced cravings for high-fat, sweets, and fast-food fats compared to placebo as early as Week 3 [33,34]. Overall, these results suggest that tirzepatide may effectively reduce the drive to eat even in the initial stages of treatment, supporting early improvements observed in our study.

Obesity continues to impact a substantial proportion of adults across multiple interconnected health domains in the US [36]. Despite the pronounced burden, access to qualified healthcare and insurance coverage for OMs remains limited in the US [37]. In our survey, 57.7% participants delayed tirzepatide initiation because they were worried about the cost of OMs, while at least 47.3% were worried that their employer-provided insurance would not cover treatment for obesity/overweight (Fig. 3b). These findings emphasize the financial burden faced by participants with obesity or overweight in the real-world, and the need for comprehensive health plans with coverage for evidence-based OMs and wellness programs, similar to other chronic diseases.

## 5 Strengths and limitations

This real-world survey combined validated PRO measures with custom questions to comprehensively capture patient insights through brief, easy-to-complete assessments that aimed to minimize participants’ burden. Findings from this study provide critical insights into the first-hand experiences of participants with obesity or overweight, identifying multiple factors that may be perceived as barriers to appropriate care and response to therapy. However, there are some limitations. Findings from our study are not generalizable and cannot be applied to all adults living with obesity in the US, such as those not on tirzepatide treatment or those using other OMs. Further, given that the focus of this survey was on individuals who were employed full-time, these results may not reflect individuals who are unemployed, underemployed, or uninsured. While electronic surveys completed by participants at their preferred location may have minimized burden, limited access to an electronic device or internet connection may have excluded some eligible participants from participation. Survey data are also captured 4–8 weeks post tirzepatide initiation and may be affected by participant recall bias. In addition, the absence of open-ended qualitative data limited our ability to capture nuanced participant experiences.

## 6 Conclusions

This survey presented a comprehensive approach to identifying the real-world disease burden, drivers, and barriers associated with the use of tirzepatide among participants with obesity or overweight.

Key takeaways.

• Participants reported high rates of internalized weight bias and low physical functioning and psychosocial well-being scores at the time of tirzepatide initiation.
• The primary motivators for initiating tirzepatide were to improve overall health and well-being, physical functioning, and self-confidence.
• Barriers such as out-of-pocket cost and uncertainties related to insurance coverage highlight the need to address additional systemic barriers that limit equitable access to effective treatment options.

## Ethical statement

This study was conducted in accordance with the ethical principles of Declaration of Helsinki and is consistent with Good Pharmacoepidemiology Practices (GPPs) and applicable laws and regulations of the country where the study was conducted. The protocol and survey materials were approved by Western Institutional Review Board-Copernicus Group Institutional Review Board (WCG IRB; IRB #20251377, dated 11-Apr-2025). All participants provided written, electronic informed consent to participate in the study. Participants received a nominal honorarium for completing the survey. Unless otherwise stated, responsibility for editorial decisions and peer review process for this article was delegated to non-author Editors or non-author Associate Editors.

## Compliance with ethics guidelines

Responsibility for editorial decisions and peer review process for this article was delegated to non-author Obesity Pillars Editors and non-author Obesity Pillars Associate Editors.

## CRediT author statement

All authors have read and agreed to the published version of the manuscript.

Theresa Hunter Gibble: Conceptualization; Design; Investigation; Roles/Writing - original draft; Writing - review & editing.

Harriet Makin: Design; Data acquisition; Formal analysis; Investigation; Roles/Writing - original draft; Writing - review & editing.

Claire Gerber: Design; Investigation; Roles/Writing - original draft; Writing - review & editing.

Catherine Bottomley: Design; Data acquisition; Investigation; Roles/Writing - original draft; Writing - review & editing.

Xuanyao He: Investigation; Roles/Writing - original draft; Writing - review & editing.

Sara Pisani: Data acquisition; Investigation; Roles/Writing - original draft; Writing - review & editing.

Michael Shepherd: Formal analysis; Investigation; Roles/Writing - original draft; Writing - review & editing.

Elizabeth Collins: Design; Data acquisition; Formal analysis; Investigation; Methodology; Roles/Writing - original draft; Writing - review & editing.

Angela Golden: Formal analysis; Investigation; Roles/Writing - original draft; Writing - review & editing.

Harold Bays: Investigation; Roles/Writing - original draft; Writing - review & editing.

## Artificial intelligence (AI)

Artificial intelligence (AI) and AI-assisted technologies were not utilized in the writing process.

## Declaration

Part of this data was presented at the OMA Annual Conference 2026.

## Funding

This study and all support for the manuscript was funded by 10.13039/100004312Eli Lilly and Company, Indianapolis, United States.

## Declaration of interest

THG, MS, CG, and XH are employees and shareholders of Eli Lilly.

HM, SP, CB and EC are employees and in some cases stockholders of Clarivate Analytics. Clarivate Analytics provides consultancy to various pharmaceutical and biotechnology companies, including Eli Lilly, the funder of this research.

AKG has served as a speaker for Lilly, Novo Nordisk, and Currax and a consultant to Lilly, Novo Nordisk, Currax, and Epitomee.

HEB's research site institution has received research grants from 89Bio, Abbvie, Allergan, Alon Medtech/Epitomee, Aligos, Altimmune, Amgen, Anji Pharma, AstraZeneca, Bioage, Biohaven, Bionime, Boehringer Ingelheim, Carmot, Chorus/Bioage, Eli Lilly, Esperion, Evidera, Fractyl, GlaxoSmithKline, Graviton, HighTide, Home Access, Horizon, Ionis, Kallyope, LG-Chem, Marea, Madrigal, Marea, Merck, Mineralys, New Amsterdam, Novartis, NovoNordisk, Pfizer, Regeneron, Satsuma, Selecta, Shionogi, Skye/Birdrock, Tern, TIMI, Veru, Viking, Vivus, Zomagen. HEB has served as a consultant (e.g., executive/national committee member and/or protocol/drug development advisor) for 89Bio, Altimmune, Amgen, Boehringer Ingelheim, Eva Pharma, Kiniksa, HighTide, Lilly, Nestle, Novo Nordisk, Regeneron, Rivus, Veru, Zomagen, ZyVersa, and serves as an editor on the Obesity Pillars board.

## Data Availability

The data that support the findings of this study are available from the corresponding author upon reasonable request.
