# Supplementary material for: Understanding reasons for initiation and experience with tirzepatide among individuals with obesity or overweight: Results from the PERCEPTIONS survey
Source: Obes Pillars. 2026 Jun 17;19:100288. doi: 10.1016/j.obpill.2026.100288 (PMC13355407; doi:10.1016/j.obpill.2026.100288)
Supplement: Multimedia component 1 [file mmc1.docx]

## **Supplementary**

### ***Table S1: Previous treatment patterns and associated weight reduction***

| **Question:** Before taking tirzepatide, have you been prescribed any other medication(s) to treat obesity/overweight? | | | |
| --- | --- | --- | --- |
|  | **I have not taken this medication** | **I have taken this medication and it did not help me to lose weight or maintain weight loss** | **I have taken this medication, and it helped me to lose weight** |
| Benzphetamine | 512 (98.8%) | 4 (0.8%) | 2 (0.4%) |
| Bupropion-naltrexone | 478 (92.3%) | 29 (5.6%) | 11 (2.1%) |
| Diethylpropion/Amfepramone | 511 (98.6%) | 2 (0.4%) | 5 (1.0%) |
| Gel capsules or fiber supplements that expand in the stomach | 493 (95.2%) | 22 (4.2%) | 3 (0.6%) |
| Liraglutide | 494 (95.4%) | 16 (3.1%) | 8 (1.5%) |
| Lorcaserin | 505 (97.5%) | 8 (1.5%) | 5 (1.0%) |
| Mazindol | 512 (98.9%) | 2 (0.4%) | 4 (0.8%) |
| A compounded formulation of a GLP-1 medication prepared by a pharmacist | 455 (87.8%) | 33 (6.4%) | 30 (5.8%) |
| Orlistat | 488 (94.2%) | 25 (4.8%) | 5 (1.0%) |
| Phendimetrazine | 506 (97.7%) | 9 (1.7%) | 3 (0.6%) |
| Phentermine | 423 (81.7%) | 50 (9.7%) | 45 (8.7%) |
| Phentermine/topiramate | 466 (90.0%) | 28 (5.4%) | 24 (4.6%) |
| Phenylpropanolamine | 511 (98.6%) | 5 (1.0%) | 2 (0.4%) |
| Semaglutide | 452 (87.3%) | 29 (5.6%) | 37 (7.1%) |
| Setmelanotide | 513 (99.0%) | 4 (0.8%) | 1 (0.2%) |
| Sibutramine | 509 (98.3%) | 6 (1.2%) | 3 (0.6%) |
| **Question:** Before taking tirzepatide, have you tried anything else to manage your weight? | | | |
|  | **I have not tried this** | **I have tried this and it did not help me lose weight or maintain weight loss** | **I have tried this and it helped me lose weight** |
| Exercising Routine | 42 (8.1%) | 257 (49.6%) | 219 (42.3%) |
| Keeping a food and/or weight diary (paper or electronic) | 118 (22.8%) | 226 (43.6%) | 174 (33.6%) |
| Consuming more healthy food and drink options | 18 (3.5%) | 234 (45.2%) | 266 (51.4%) |
| Eating diet products | 136 (26.3%) | 252 (48.6%) | 130 (25.1%) |
| Followed a special diet | 125 (24.1%) | 203 (39.2%) | 190 (36.7%) |
| Tracking physical activity or exercise (e.g. through a smartwatch) | 132 (25.5%) | 223 (43.1%) | 163 (31.5%) |
| Mindfulness or meditation | 302 (58.3%) | 162 (31.3%) | 54 (10.4%) |
| Joining a commercial weight loss program (e.g., Weight Watchers, Noom, Jenny Craig, etc.) | 265 (51.2%) | 132 (25.5%) | 121 (23.4%) |
| Workplace wellness programs | 361 (69.7%) | 110 (21.2%) | 47 (9.1%) |
| Non-workplace wellness programs | 374 (72.2%) | 95 (18.3%) | 49 (9.5%) |
| Cognitive behavioral therapy or other behavioral therapies | 433 (83.6%) | 66 (12.7%) | 19 (3.7%) |

### ***Table S2. Ease of use of tirzepatide at baseline as assessed by SQAAQ***

| **Full sample (n=518)** | | | | | | | |
| --- | --- | --- | --- | --- | --- | --- | --- |
|  | **Strongly disagree** | **Disagree** | **Slightly disagree** | **Neither agree nor disagree** | **Slightly agree** | **Agree** | **Strongly agree** |
| 1. Easy to learn how to use | 7 (1.4%) | - | 6 (1.2%) | 12 (2.3%) | 26 (5%) | 166 (32%) | 301 (58.1%) |
| 2. Easy to unlock | 8 (1.5%) | 2 (0.4%) | 3 (0.6%) | 33 (6.4%) | 23 (4.4%) | 149 (28.8%) | 300 (57.9%) |
| 3. Easy to hold in hand while injecting dose | 6 (1.2%) | 2 (0.4%) | 8 (1.5%) | 18 (3.5%) | 33 (6.4%) | 151 (29.2%) | 300 (57.9%) |
| 4. Easy to inject dose | 5 (1%) | 3 (0.6%) | 10 (1.9%) | 9 (1.7%) | 16 (3.1%) | 171 (33%) | 304 (58.7%) |
| 5. Easy to know dose is complete | 5 (1.0%) | 3 (0.6%) | 20 (3.9%) | 6 (1.2%) | 33 (6.4%) | 163 (31.5%) | 288 (55.6%) |
| 6. Easy to store device in refrigerator | 4 (0.8%) | - | 2 (0.4%) | 7 (1.4%) | 16 (3.1%) | 156 (30.1%) | 333 (64.3%) |
| 7. Easy to remove needle shield/cover | 4 (0.8%) | - | 5 (1.0%) | 17 (3.3%) | 23 (4.4%) | 157 (30.3%) | 312 (60.2%) |
| 8. Easy to pick up | 3 (0.6%) | 1 (0.2%) | - | 12 (2.3%) | 16 (3.1%) | 148 (28.6%) | 338 (65.3%) |
| 9. Overall easy to use | 3 (0.6%) | 1 (0.2%) | 5 (1.0%) | 5 (1.0%) | 14 (2.7%) | 164 (31.7%) | 326 (62.9%) |
| 10. Device is stable against skin during injection | 3 (0.6%) | - | 5 (1.0%) | 21 (4.1%) | 21 (4.1%) | 169 (32.6%) | 299 (57.7%) |
| 11. Confident in ability to use the dose | 4 (0.8%) | 4 (0.8%) | 7 (1.4%) | 12 (2.3%) | 26 (5.0%) | 157 (30.3%) | 308 (59.5%) |
| 12. Confident of dose is completion | 4 (0.8%) | 2 (0.4%) | 14 (2.7%) | 8 (1.5%) | 30 (5.8%) | 165 (31.9%) | 295 (56.9%) |
| **First dose with auto-injector/self-injector (n=351)** | | | | | | | |
| 1. Easy for me to learn how to use | 4 (1.1%) | - | 2 (0.6%) | 7 (2.0%) | 13 (3.7%) | 101 (28.8%) | 224 (63.8%) |
| 2. Easy for me to unlock | 4 (1.1%) | 1 (0.3%) | 1 (0.3%) | 7 (2.0%) | 10 (2.8%) | 94 (26.8%) | 234 (66.7%) |
| 3. Easy for me to hold in my hand when I inject dose | 3 (0.9%) | - | 2 (0.6%) | 5 (1.4%) | 16 (4.6%) | 91 (25.9%) | 234 (66.7%) |
| 4. Easy to inject my dose | 2 (0.6%) | 1 (0.3%) | 3 (0.9%) | 3 (0.9%) | 8 (2.3%) | 104 (29.6%) | 230 (65.5%) |
| 5. Easy for me to know that my dose is complete | 3 (0.9%) | 2 (0.6%) | 13 (3.7%) | 2 (0.6%) | 25 (7.1%) | 99 (28.2%) | 207 (59.0%) |
| 6. Easy to store device in refrigerator | 3 (0.9%) | - | 2 (0.6%) | 3 (0.9%) | 10 (2.8%) | 97 (27.6%) | 236 (67.2%) |
| 7. Easy to remove needle shield/cover | 2 (0.6%) | - | 5 (1.4%) | 10 (2.8%) | 14 (4.0%) | 100 (28.5%) | 220 (62.7%) |
| 8. Easy to pick up | 2 (0.6%) | 1 (0.3%) | - | 4 (1.1%) | 10 (2.8%) | 88 (25.1%) | 246 (70.1%) |
| 9. Overall easy to use | 2 (0.6%) | - | 2 (0.6%) | 3 (0.9%) | 7 (2.0%) | 93 (26.5%) | 244 (69.5%) |
| 10. Device is stable against skin during injection | 2 (0.6%) | - | 2 (0.6%) | 2 (0.6%) | 12 (3.4%) | 109 (31.1%) | 224 (63.8%) |
| 11. Confident in ability to use the dose | 2 (0.6%) | 1 (0.3%) | 4 (1.1%) | 2 (0.6%) | 15 (4.3%) | 101 (28.8%) | 226 (64.4%) |
| 12. I am confident my dose is complete | 2 (0.6%) | 2 (0.6%) | 9 (2.6%) | 4 (1.1%) | 21 (6.0%) | 108 (30.8%) | 205 (58.4%) |
| **First dose injected from a vial (n=167)** | | | | | | | |
| 1. Easy for me to learn how to use | 3 (1.8%) | - | 4 (2.4%) | 5 (3.0%) | 13 (7.8%) | 65 (38.9%) | 77 (46.1%) |
| 2. Easy for me to unlock | 4 (2.4%) | 1 (0.6%) | 2 (1.2%) | 26 (15.6%) | 13 (7.8%) | 55 (32.9%) | 66 (39.5%) |
| 3. Easy for me to hold in my hand when I inject dose | 3 (1.8%) | 2 (1.2%) | 6 (3.6%) | 13 (7.8%) | 17 (10.2%) | 60 (35.9%) | 66 (39.5%) |
| 4. Easy to inject my dose | 3 (1.8%) | 2 (1.2%) | 7 (4.2%) | 6 (3.6%) | 8 (4.8%) | 67 (40.1%) | 74 (44.3%) |
| 5. Easy for me to know that my dose is complete | 2 (1.2%) | 1 (0.6%) | 7 (4.2%) | 4 (2.4%) | 8 (4.8%) | 64 (38.3%) | 81 (48.5%) |
| 6. Easy to store device in refrigerator | 1 (0.6%) | - | - | 4 (2.4%) | 6 (3.6%) | 59 (35.3%) | 97 (58.1%) |
| 7. Easy to remove needle shield/cover | 2 (1.2%) | - | - | 7 (4.2%) | 9 (5.4%) | 57 (34.1%) | 92 (55.1%) |
| 8. Easy to pick up | 1 (0.6%) | - | - | 8 (4.8%) | 6 (3.6%) | 60 (35.9%) | 92 (55.1%) |
| 9. Overall easy to use | 1 (0.6%) | 1 (0.6%) | 3 (1.8%) | 2 (1.2%) | 7 (4.2%) | 71 (42.5%) | 82 (49.1%) |
| 10. Device is stable against skin during injection | 1 (0.6%) | - | 3 (1.8%) | 19 (11.4%) | 9 (5.4%) | 60 (35.9%) | 75 (44.9%) |
| 11. Confident in ability to use the dose | 2 (1.2%) | 3 (1.8%) | 3 (91.8%) | 10 (6.0%) | 11 (6.6%) | 56 (33.5%) | 82 (49.1%) |
| 12. I am confident my dose is complete | 2 (1.2%) | - | 5 (3.0%) | 4 (2.4%) | 9 (5.4%) | 57 (34.1%) | 90 (53.9%) |

Auto‑injector and vial subgroups are mutually exclusive as defined by first dose device, and sample sizes are n=351 and n=167, respectively. SQAAQ assesses ease of use using a 7-point Likert-type response scale to indicate level of agreement with each item (strongly disagree, disagree, slightly disagree, neither agree nor disagree, slightly agree, agree, strongly agree). Higher scores indicate greater agreement (ease of use). No recall period is provided. n, number of participants; SQAAQ, Subcutaneous Administration Assessment Questionnaire.
